# Prevalence of Dementia and Cognitive Complaints in the Context of High Cognitive Reserve: A Population-Based Study

**DOI:** 10.1371/journal.pone.0138818

**Published:** 2015-09-21

**Authors:** Magali Perquin, Nico Diederich, Jessica Pastore, Marie-Lise Lair, Saverio Stranges, Michel Vaillant, on behalf of the MemoVie Group

**Affiliations:** 1 Department of Population Health, Luxembourg Institute of Health (LIH), Strassen, Luxembourg; 2 Department of Neurology, Centre Hospitalier de Luxembourg (CHL), Luxembourg, Luxembourg; 3 Competence Center in Methodology and Statistics, Luxembourg Institute of Health (LIH), Strassen, Luxembourg; Cardiff University, UNITED KINGDOM

## Abstract

**Objectives:**

This study aimed to assess the prevalence of dementia and cognitive complaints in a cross-sectional sample of Luxembourg seniors, and to discuss the results in the societal context of high cognitive reserve resulting from multilingualism.

**Methods:**

A population sample of 1,377 people representative of Luxembourg residents aged over 64 years was initially identified via the national social insurance register. There were three different levels of contribution: full participation in the study, partial participation, and non-participation. We examined the profiles of these three different samples so that we could infer the prevalence estimates in the Luxembourgish senior population as a whole using the prevalence estimates obtained in this study.

**Results:**

After careful attention to the potential bias and of the possibility of underestimation, we considered the obtained prevalence estimates of 3.8% for dementia (with corresponding 95% confidence limits (CL) of 2.8% and 4.8%) and 26.1% for cognitive complaints (CL = [17.8–34.3]) as trustworthy.

**Conclusion:**

Based on these findings, we postulate that high cognitive reserve may result in surprisingly low prevalence estimates of cognitive complaints and dementia in adults over the age of 64 years, which thereby corroborates the longer disability-free life expectancy observed in the Luxembourg population. To the best of our knowledge, this study is the first to report such Luxembourgish public health data.

## Introduction

Beyond the consideration of life expectancy is that of life expectancy without disability, a major public health concern in developed countries. In the case of neurodegenerative diseases, all approaches, pharmacological or others that are able to delay the loss of autonomy are of great interest. Indeed, neurodegenerative diseases represent a growing public health problem: The World Health Organization has estimated that 35 million people worldwide have dementia, and, with the ageing of the world’s population, this number is expected to triple by 2050 [1]. Alzheimer’s disease (AD) is the most common form of dementia, representing 67% of cases [2]. According to US official death certificates, the proportion of deaths resulting from AD increased by 68% between 2000 and 2010 [3].

In the context of dementia, the concept of cognitive reserve has been of huge interest in more recent years, especially as, so far, pharmacological strategies have remained unsuccessful. Cognitive reserve has been defined as brain resource that is developed through lifelong, challenging cognitive activities and that protects individuals from clinical signs of cognitive decline [4–11]. Moreover, cognitive decline has been shown to be strongly associated with earlier subjective memory complaints (SMCs), which suggests that individuals with SMCs have a higher risk of developing dementia [12, 13]. Notably, the literature describes SMCs or cognitive complaints as “the expression of the perceived experience of everyday forgetfulness” [14], an aspect which has gained increasing interest. Even if certainly disputable, SMCs have been more widely studied as a predictor for cognitive decline and/or dementia, than as a risk marker of an early manifestation of dementia pathology, which is known to be associated to an anosognosia state, displaying a lack of awareness of the decline in memory. As such, a substantial importance is placed on SMCs with Peterson’s group recommendations [15], which has included them in the elaboration of mild cognitive impairment (MCI) diagnosis.

The present manuscript provides, for the first time, reliable prevalence estimates of dementia and of cognitive complaints among seniors from Luxembourg. The study sample used came from the MemoVie cohort [16], and was selected to be representative of this population. Due to substantial refusals, we further dissected the characteristics of participation in order to demonstrate that, despite these restrictions, robust and trustworthy findings were produced. The obtained prevalence estimates of dementia and cognitive complaints are discussed in the particular context of a high cognitive reserve conferred by multilingualism.

Indeed, multilingualism is highly prevalent in Luxembourg, since the linguistic situation there is characterized by practice and recognition of three official languages. Multilingualism is all the stronger as these languages are taught from the youngest age and, permanently practiced according to the context of life. This makes the multilingual situation a bit different from that of other countries, where multilingualism is more associated with geographic areas. In these conditions, speaking a fourth or fifth language is not uncommon, as long as it comes from a foreign family heritage. Hence, contextual cognitive reserve-that we have already described (8)- being high, we may reasonably raise the question of the hypothetical effect that this situation could have on prevalence estimates of dementia and cognitive complaints. This issue is even more relevant given that previous studies assumed that contributing to cognitive reserve, lifelong multilingualism (8) or bilingualism (39) provides protection against cognitive impairment or Alzheimer’s disease.

## Participants and Methods

### Population, study design, and sampling frame

The MemoVie study was originally designed to explore the national prevalence of AD and MCI. A cohort representative of the senior population of Luxembourg was set up as a baseline. Stratified by age groups and gender, potential participants were randomly selected from the General Inspectorate of Social Security (IGSS) register, which covers about 97% of the total population. By selecting Luxembourg residents aged over 64 years on January 1, 2008 from this register, we were able to build up the initial sampling frame.

The study offers a standardized stepwise protocol (at home participant or research centre) including extensive neuropsychological evaluation, medical history investigation, and clinical examination in case of suspicion of cognitive impairment. The exhaustive criteria and decision algorithm leading to diagnosis, have been described in detail elsewhere [16]. The cognitive evaluation included the extended version of the Consortium to Establish a Register for Alzheimer’s Disease-Neuropsychological Battery (CERAD-NP-plus; [17]), as well as nine additional tests, comprising the Beck Anxiety Inventory (BAI; Beck et al., 1988) and the Geriatric Depression Scale (GDS; [18]), which assess current or previous anxiety and depression. Standardisation between investigators was guaranteed by an external expertise who reviewed all cases and provided validated diagnoses. Absence of inter-investigator variation has been statistically verified.

### Sample size and power estimation

The sample size was calculated based on the estimation of a 20% prevalence of MCI [17,18]. This made it possible to predict the prevalence of both AD and overall dementia, both expected to be far less than 20% (about 3 to 6% and 6 to 9% respectively, depending on the source [19–21]). The sample size calculation considered a precision of 2.5% around the calculated prevalence estimates and a 95% confidence interval to compute the sample size of 983 people. Assuming a hypothetical refusal rate of 40%, the sample needed to include at least 1,377 participants.

### Sampling

According to the National Institute of Statistics and Economic Studies of the Grand Duchy of Luxembourg (Statec), the national population aged 65 years and over was 66,000 at the beginning of the study. This constituted the sampling frame.

The sampling plan was stratified and randomized with an allocation probability (chance to be selected is equal for all individuals of the same age category and gender) proportional to size (of the population) without replacement (the same individual could not be selected twice). The selection probability for unit *i* (for example 65-69y) in stratum *h* (for example men) equals *n*_*h*_ *Z*_*hi*_, where *n*_*h*_ is the sample size for stratum *h*, and *Z*_*hi*_ is the relative size of unit *i* in stratum *h*. The relative size equals *M*_*hi*_ */M*_*h*_, which is the ratio of the size measure for unit *i* in stratum *h (M*_*hi*_ = *number of men aged 65-69y)* to the total of all size measures for stratum *h (M*_*h*_ = number of men) [22].

### Prevalence calculation

The prevalence estimates of dementia and cognitive complaints were evaluated by using the initial sampling plan described above. The probability to be sampled n_h_Z_hi_ was calculated for each individual based on the size of each age and gender strata in the sample. It was included as a weighting parameter in the calculation of the prevalence ∑(x_i_*n_h_Z_hi_), which was estimated by summing up data on all patients. To include a finite population correction in Taylor series variance estimation, the size of each age and gender strata in the target population was also entered in the analyses in order to calculate the Wald 95% confidence interval, and thereby extrapolate the proportion of dementia to the target population. The surveyfreq procedure of the statistical software SAS System V9.3 (SAS Institute, Cary, NC) was used.

### Approach of the invited people refusing a full participation: the partial and non-participants

**Fig 1:**
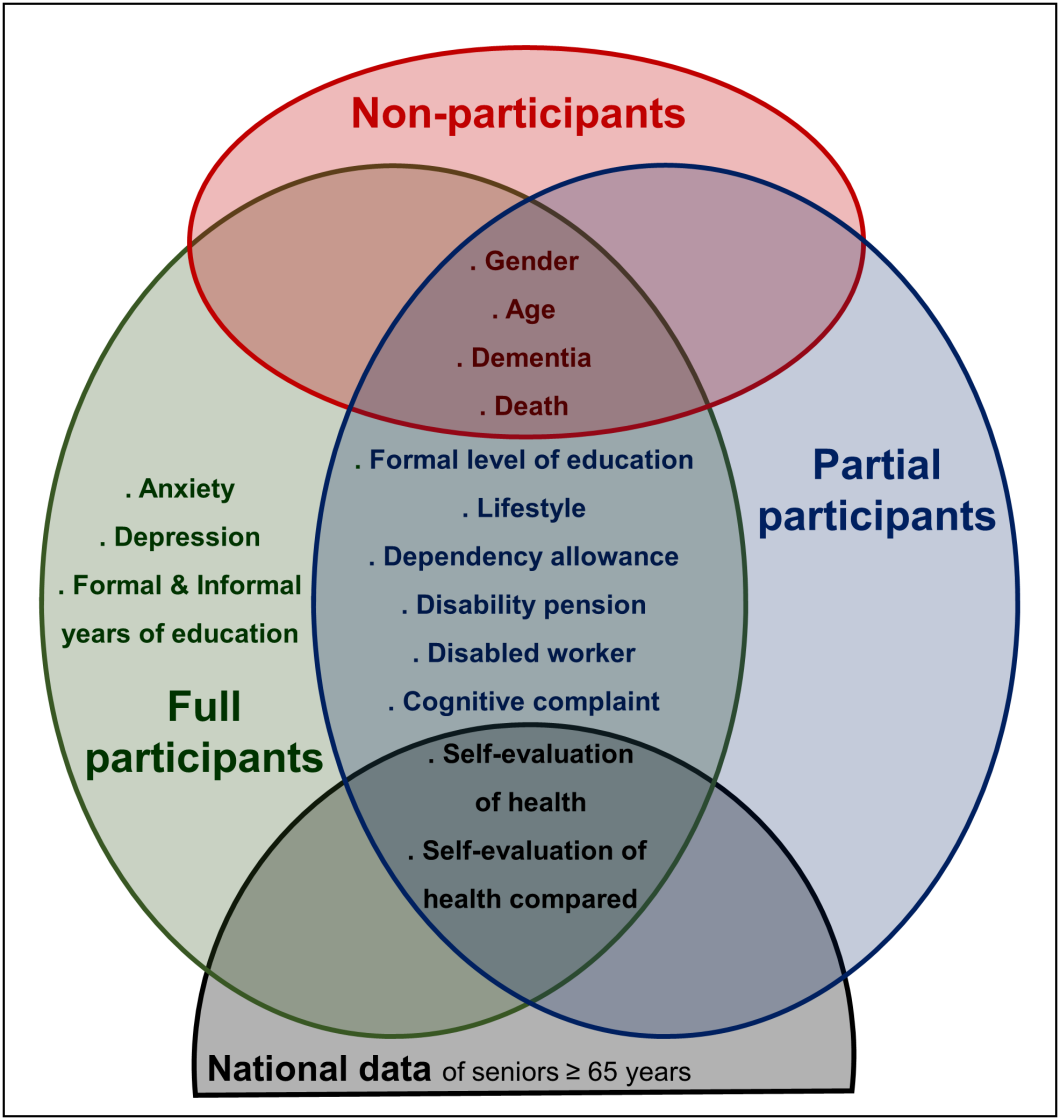
Set of data according to the groups of population.

Age and gender information for the 1,377 people invited to participate was provided by the IGSS register. More people (68.2%) than expected (40.0%) declined to participate, which could have jeopardized the representativeness of the sample. We therefore collected a minimum data set from those reluctant to participate, which allowed for (1) the characterization of this population, and (2) a comparison with participants. Individuals that refused to participate were asked to complete a short phone interview administered by a trained investigator. This interview collected information about socio-cultural variables (education, life style, and habitat), autonomy (if receiving financial assistance for disability or loss of autonomy), cognitive complaints (estimated using scores obtained on the QPC (Questionnaire de Plainte Cognitive [23]) and also included a subjective assessment of health. Moreover, information on diagnosis of dementia was obtained by family declaration during the preliminary phone contact dedicated to the organization of appointments. Only “formal diagnosis” made within classical care system (by personal physicians), and reported by caregiver or family member was recorded. Information about the death of the invited person was also collected through family contact during the phone call, as well as actively researched through public declaration of death for drop outs. Close relatives' answers about both conditions (dementia or death) were not doubtful, and we were assured of collecting reliable data. Fig 1 depicts the dataset available in the different groups of population, and the existing overlap.

### Comparison between full participants, partial participants, and non-participants

The analyses provided results on the distribution of people who consented to take part to the study (the full participants, *n* = 286), people who agreed to answer a short interview (the partial participants, *n* = 152), and people who declined to participate (the non-participants, *n* = 939). Gender, dementia, and mortality analyses on the three groups were performed using Chi-square test, and age was studied using the Kruskal-Wallis test. The Nemenyi test was used for pairwise multiple comparisons to locate the source of significance. Apart from these, no other data was available on the group of people who refused to participate.

However, the short interview accepted by 152 people supplied us with more specific data that were useful for further comparisons between “full” and “partial” participants. The available items in the short interview were on education, lifestyle, dependency, disability pension, status as a disabled worker, self-evaluation of health, self-evaluation of health compared with counterparts, and cognitive complaints. Chi-square test and Fisher's exact test were used, as appropriate, for the analyses of these data.

### Comparison between full participants, partial participants, and national data

Data obtained from this study were compared with information reported by national and/or European institutes. The comparison was performed according to the best temporal adjustment. The results were visualized by graphical comparisons and analyzed using a Chi-square test.

### Analysis of cognitive complaints

The Chi-square test was used to analyze the association between cognitive complaints and gender among the entire “full and partial” participants group, as well as between cognitive complaints and anxiety or depression among the full participants only. The Mann-Whitney test was used to examine the association between cognitive complaints and age or education.

A *p*-value of <0.05 was considered as statistically significant. All tests were two-tailed. Statistical analyses were carried out with the statistical package SAS System version 9.3 (SAS Institute, Cary, North Carolina, USA). In some case, the lack of some data resulted in different sample sizes within the various analyses performed. Data is available in S1 Dataset.

### Ethics

Ethical and legal approval for the MemoVie study were obtained from the National Research Ethics Committee (CNER) and the National Commission for Data Protection (CNPD) in Luxembourg, respectively. Written consent was obtained from participants and records were kept in a secure place. People could refuse to participate in the full study, and verbally consented to answer a very short questionnaire over the phone. Protocol, informed consent content, recording of agreement, and documentation of participants' consent were also approved. Individual identities were made immediately anonymous after the call by applying a random calculation, which generated a bar code that was recognizable by a scanner only. The person responsible for the data entry, further data management steps, as well as the team project was different from the person in charge of the call to guarantee the confidentiality of data.

## Results and Discussion

### Participation rate

**Fig 2:**
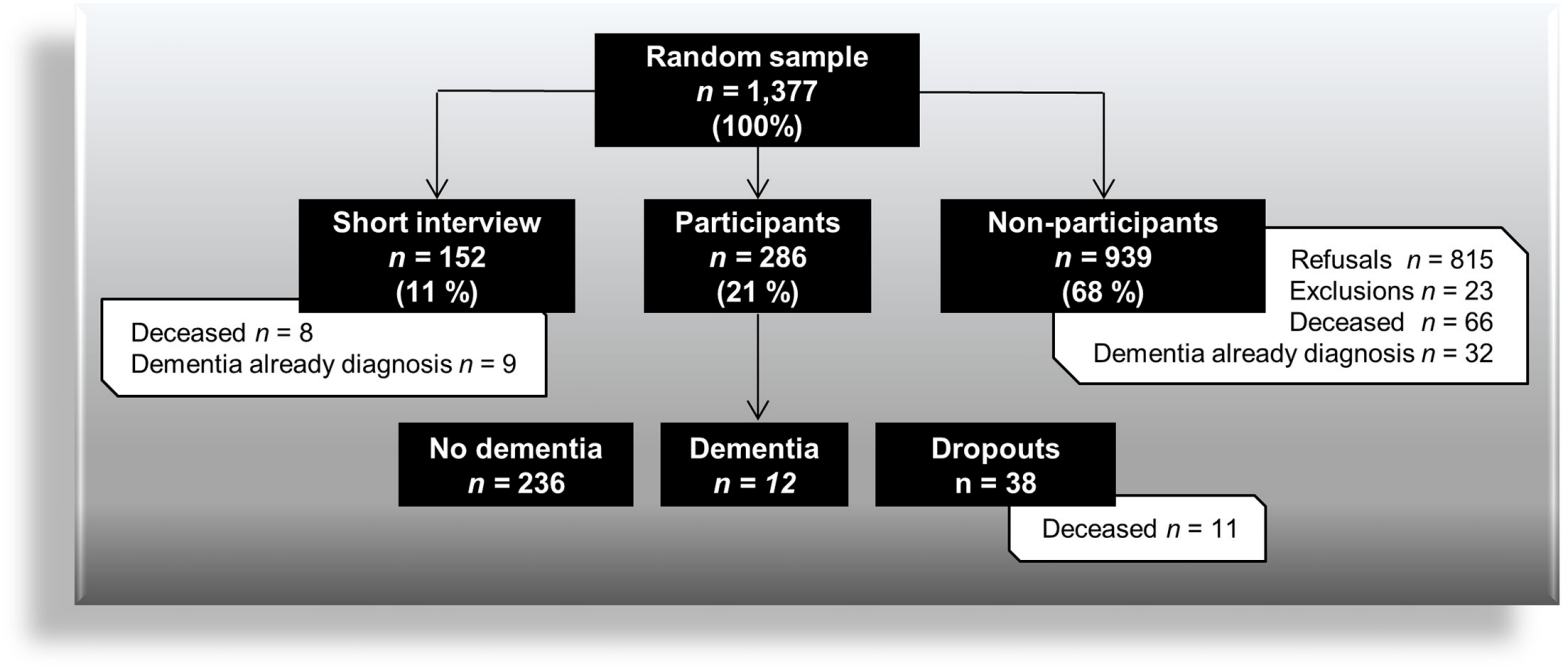
Flow chart showing participation rates and prevalence of dementia observed in the MémoVie cohort study.

Of the 1,377 individuals sampled, 438 joined the study; 286 of them (20.8%) completed the whole study (full participation), and 152 (11.0%) accepted partial contribution consisting of the phone interview (partial participation). Finally, 939 subjects (68.2%) declined to participate (non-participation) (Fig 2).

**Full participation** was initially offered by 286 people, 236 of whom (82.5%, or 17.1% of the total) were classified as dementia-free, 12 (4.2%, or 0.9% of the total) showed a syndrome of dementia, and 38 (13.3%, or 2.7% of the total) dropped out during the inclusion period, which included 11 (3.8%, or 0.8% of the total) who died. **Partial participation** was initially offered by 253 people (18.4%), but finally 40% of them changed their mind and refused to participate. Eight participants from this group (5.3%, or 0.6% of the total) died, and 9 (5.9%, or 0.6% of the total) were already diagnosed with dementia. **Non-participation** was constituted by 66 individuals (7.0%, or 4.8% of the total) who died during the inclusion period, 32 individuals (3.4%, or 2.3% of the total) previously diagnosed with dementia and 23 persons (2.4%, or 1.7% of the total) who were excluded because of language incompatibility (*n* = 6) as well as loss of eyesight or hearing (*n* = 17), which made neuropsychological assessment of cognitive function impossible. Furthermore, we obtained the reasons for refusal in 90.4% of the cases (737 out of 815 individuals): 61.7% of people (*n* = 455) were “*not interested*” and “*just want to be left alone*”; 17.5% of individuals (*n* = 129) had too precarious health to be available for such a study; and 16.3% (*n* = 120) claimed that they were too busy travelling and devoting themselves to leisure (which indicated a rather balanced population: both extremes, i.e., particularly healthy and particularly frail people may not have been included); 3% (*n* = 22) refused for familial reasons, and 1.5% (*n* = 11) used very low mobility as an explanation for refusal. In addition, it should be noted that the study topic may have been partly responsible for the low rate of participation and dropout rate, since the fear of having to face a potential unexpected memory problem was not an attractive prospect.

### Characterization of the 3 types of population

We compared the three types of subjects (full participants, partial participants, and non-participants) on the common variables of gender, age, dementia, and death. Table 1 summarizes the distribution of the three populations on these measures.

**Table 1:** Distribution of data available for the three types of population.

Variable	Full participation	Partial participation	Non-participation	*p*-value
	(*n* = 286)	(*n* = 152)	(*n* = 939)	
**Gender, male, *n* (%)**	124 (43.4)	62 (40.8)	382 (40.7)	*p* = 0.72^a^
**Age, y, mean (SD)**	73.6 (5.7)	74.9 (6.1)	76.2 (7.3)	*p* < 0.0001^b^
**Dementia, *n* (%)**	12 (4.2)	9 (5.9)	32 (3.4)	*p* = 0.31^a^
**Death, *n* (%)**	11 (3.8)	8 (5.3)	66 (7.0)	*p* = 0.13^a^

^a^: the *p*-value was derived from Chi-squared test

^b^: the *p*-value was derived from Kruskal-Wallis test

The female-to-male ratio, as well as dementia and death rates were equally distributed between the 3 groups. On the other hand, there was a significant between-group difference in mean age. Post-hoc pairwise comparisons revealed that this main effect stemmed from a significant difference between full-participants and non-participants (2.6 (95%CI = [1.5;3.7]) years difference in mean age). The mean age of the partial participants fell between that of these two groups, and was not significantly different from either the full-participants (1.3 (95%CI = [-0.3;2.9]) years difference in mean age) or the non-participants (-1.3 (95%CI = [-2.7;0.1]) years difference in mean age).

### Who were the reluctant participants?

The distribution of the **education** levels (Table 2) was significantly different between people who adhered to the full protocol and partial participants. Full participants were more educated (58.7% with secondary education and 16.8% with higher or academic education) than the others (50.0% with secondary education and 3.7% with higher or academic education). Moreover, the percentage of individuals who had not exceeded primary school was 46.3% for partial versus 24.5% for full participants.

**Table 2:** Characteristics for both full and partial participants.

	Partial participants (*n* = 152)		Full participants (*n* = 286)		*p*-value
**Education**, *n* (%)					
No schooling	8	(5.88)	0	(0.00)	<0.0001^a^
Primary school	55	(40.44)	70	(24.48)	
Secondary 1st cycle	42	(30.88)	123	(43.01)	
Secondary 2nd cycle	26	(19.12)	45	(15.73)	
Higher education (short)	1	(0.74)	31	(10.84)	
Higher education—University	4	(2.94)	17	(5.94)	
Other	0	(0.00)	0	(0.00)	
Do not know	0	(0.00)	0	(0.00)	
Missing	16		0		
**Lifestyle**, *n* (%)					
Single	41	(30.37)	76	(26.57)	0.02^a^
Couple	73	(54.07)	175	(61.19)	
Familial cohabitation	20	(14.81)	22	(7.69)	
Non-familial cohabitation	1	(0.74)	13	(4.55)	
Do not know	0	(0.00)	0	(0.00)	
Missing	17	(11.18)	0	(0.00)	
**Dependency allowance**, *n* (%)					
No	118	(87.41)	248	(87.32)	0.17^b^
Yes	14	(10.37)	35	(12.32)	
Request in process	0	(0.00)	0	(0.00)	
Do not know	3	(2.22)	1	(0.35)	
Missing	17	(11.18)	2	(0.70)	
**Disability pension**, *n* (%)					
No	114	(84.44)	253	(89.08)	0.16^b^
Yes	20	(14.81)	31	(10.92)	
Do not know	1	(0.74)	0	(0.00)	
Missing	17	(11.18)	2	(0.70)	
**Disabled worker**, *n* (%)					
No	132	(97.78)	278	(97.89)	0.08^b^
Yes	1	(0.74)	6	(2.11)	
Request in process	0	(0.00)	0	(0.00)	
Do not know	2	(1.48)	0	(0.00)	
Missing	17	(11.18)	2	(0.70)	
**Self-evaluation of health**, *n* (%)					
Very good	18	(13.33)	24	(9.30)	0.055^b^
Good	56	(41.48)	147	(56.98)	
Intermediate	48	(35.56)	72	(27.91)	
Bad	11	(8.15)	12	(4.65)	
Very bad	1	(0.74)	2	(0.78)	
Do not know	1	(0.74)	1	(0.39)	
Missing	17	(11.18)	28	(9.79)	
**Self-evaluation of health compared** *, *n* (%)					
Worse	7	(5.38)	19	(7.36)	<0.0001^a^
As good as	55	(42.31)	97	(37.60)	
Better	35	(26.92)	130	(50.39)	
Do not know	33	(25.38)	12	(4.65)	
Missing	22	(14.47)	28	(9.79)	
**Questionnaire of cognitive complaints** ^#^, *n* (%)					
Complaint	34	(22.37)	71	(24.83)	0.86^a^
No complaint	102	(67.11)	204	(71.33)	
Missing	16	(10.53)	11	(3.85)	

* to people of a similar age

^a^ *p*-values derived from Chi-squared tests;

^b^ *p*-values derived from Fisher's exact tests.

^#^ used with a cut-off based on the algorithm: Total score ≥ 3 or score for question 5 = 1 or score of the sum of questions A, 4, 5, 7, 8 ≥ 2.

The distribution of lifestyle (Table 2) differed significantly between full and partial participants. While the majority of the subjects in both populations lived as a couple, this was more common in full participants than partial participants (61.2% vs. 54.1%). However, familial cohabitation was more common in partial participants (14.8% vs. 7.7%). Nevertheless, this did not seem to reflect a greater dependence of these people. Indeed, both populations seemed to have the same level of autonomy, since dependency allowance, disability pension, and earlier disabled worker status were not significantly different between the two groups.

Both full and partial participants appeared to have a rather good opinion of their health. When they were asked to self-evaluate their health in comparison with people of similar age, the full-participants were twice as likely to respond with the optimistic answer “better” than the partial participants (Table 2). Partial participants seemed less able to make this comparison (25.4% responded “do not know” vs. 4.6% for full participants). Thereby, partial participants were apparently less surrounded by persons of their age. Indeed, they lived less frequently “in couple” or in “non-familial cohabitation” and more frequently in “familial cohabitation” or “single” (Table 2).

Finally, even if people’s feelings about their own health were significantly different between the two groups, with possibly slightly greater optimism in full participants, this aspect was not reflected by the cognitive complaints reported (Table 2, *p* = 0.86). Noteworthy is that cognitive complaints have been well described to be a strong indication of the current or upcoming memory impairment [24–26], and are even part of the criteria defined by Petersen [27] for the MCI status.

### Comparison between the overall participants (i.e., full and partial participants) and the Luxembourgish national data

It seemed relevant to extend the comparison between the population of participants (full and partial), from which results will be calculated, and the available national data on seniors. For parameters on which the full and partial participants were not different (age and gender), data for all participants (full and partial) were compared with the national data on seniors, whereas, on parameters for which differences were found between full and partial participants (lifestyle and self-evaluation of health), the national data on seniors were compared to data for specific groups (full participants and all participants) separately.

**Fig 3:**
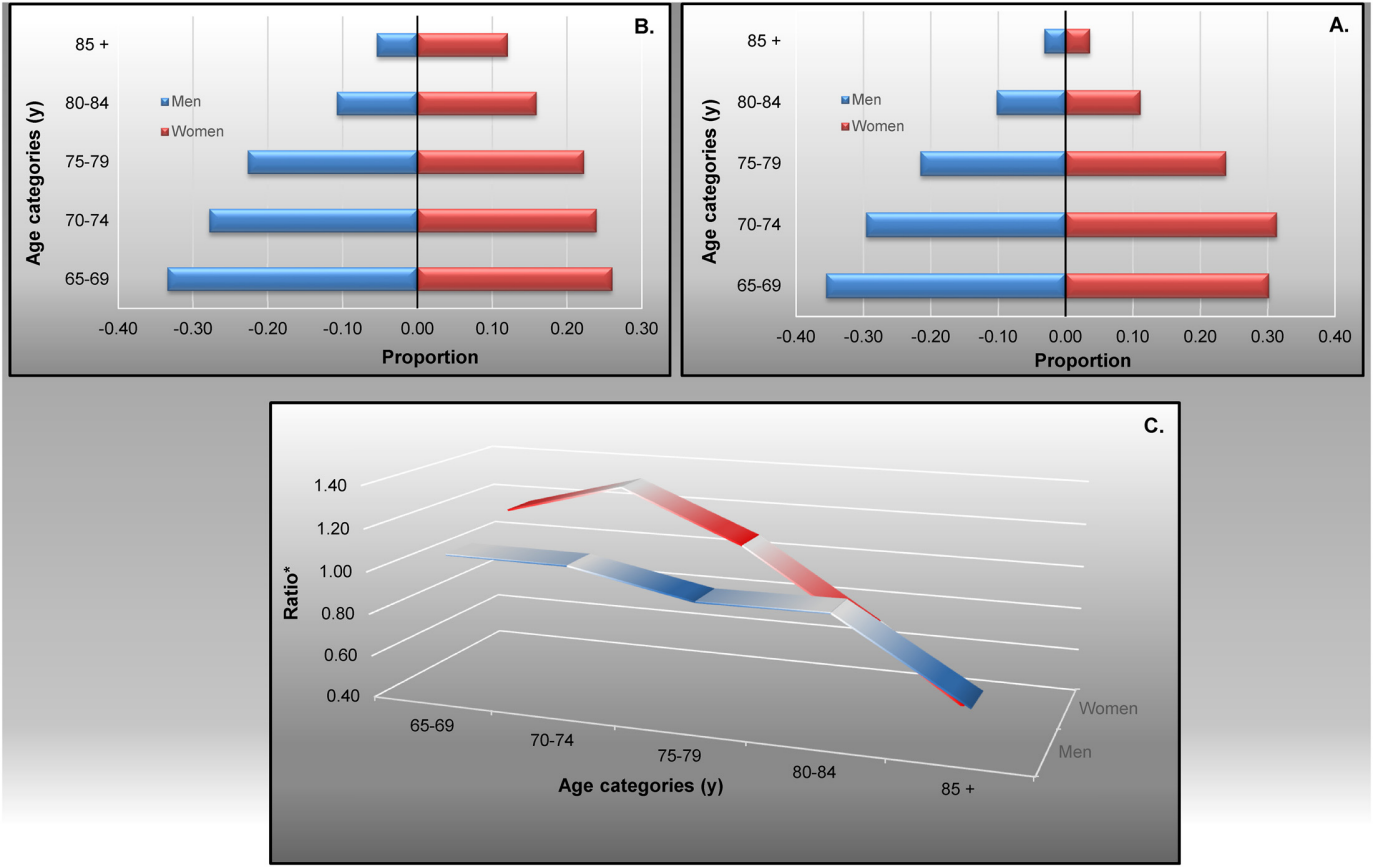
Population pyramid of the overall participants (A) and of seniors from Luxembourg (B): Comparison by gender and age (C). (A) Overall participants: per age group and gender, numbers compared to the total amount of subjects over 64 years old. (B) Luxembourgish seniors: per age group and gender, numbers compared to the total amount of subjects over 64 years old. (C) Representativeness depending on age groups and gender: the overall participants compared with the Luxembourgish seniors. Overall participants include full-participants and partial participants to the MemoVie study (*n* = 438). National statistics 2006, from: http://www.statistiques.public.lu/stat/TableViewer/tableView.aspx?ReportId=385&IF_Language=eng&MainTheme=2&FldrName=1 (*n* = 66,000)

The population pyramid structures of the overall participants (Fig 3A) and the national data on seniors (Fig 3B) were similar. They almost completely overlapped for men, and were analogous for women until the age of 79 years (with a slight over-representation of the age group 70–74 years in the overall participants). From then on, and especially from 85 years old, women were less well represented in the overall participants. Fig 3C illustrates the ratios between proportions of subjects in the same age category from the overall participants and the national data on seniors, which, when close to 1, indicates a perfect representativeness between groups.

Over- and under-representation of women and men from the overall participants, according to age category are further described in Fig 3C, and were still acceptable below 85 years of age.

**Fig 4:**
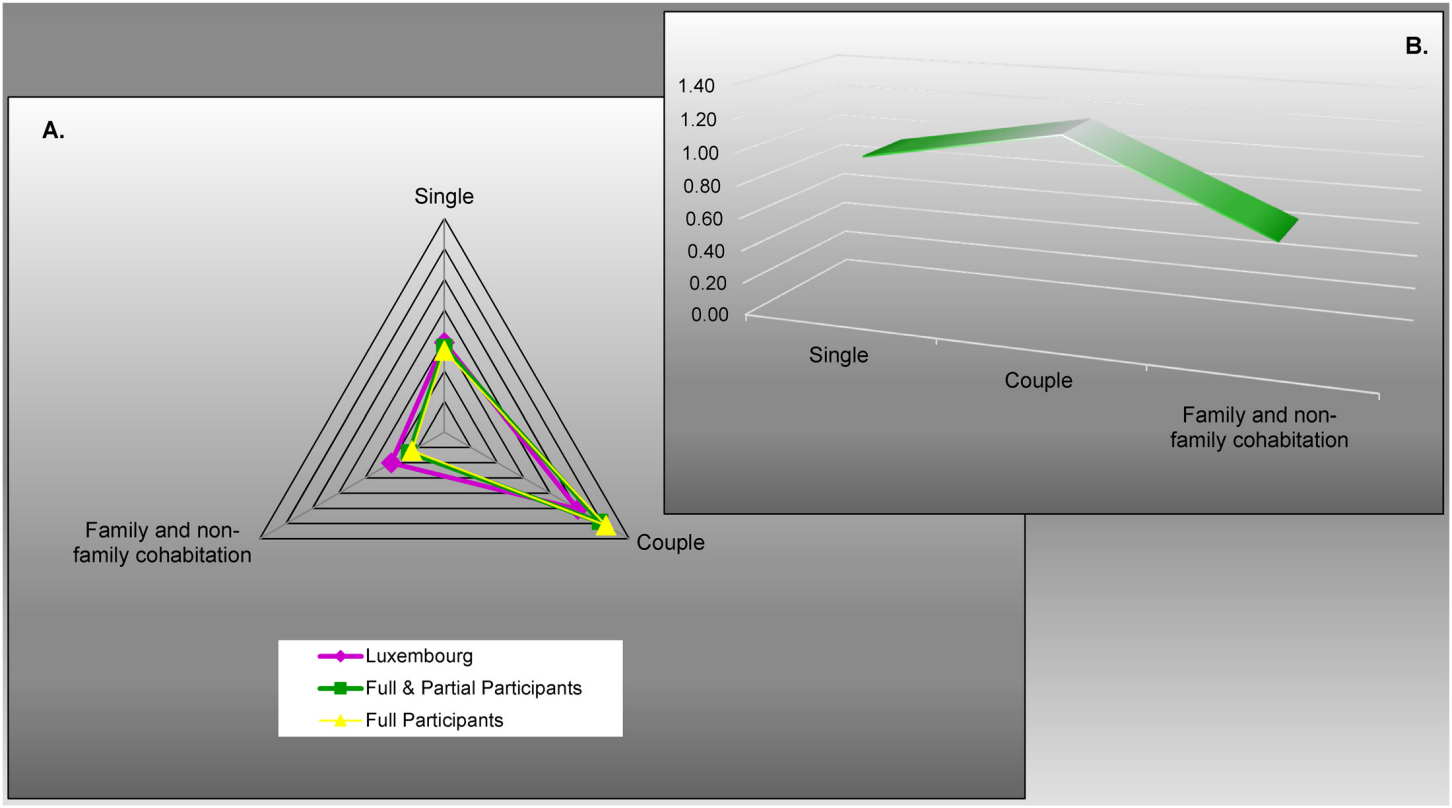
Answers supplied for “lifestyle.” (A) Lifestyle distribution depending on population. (B) Representativeness depending on lifestyle: the overall participants compared to the Luxembourgish seniors. From the full-participants (*n* = 286), the overall participants (*n* = 421) and from the general population ≥ 65 years of Luxembourg (source: Statec 2001, *n* = 57,230). For this criteria, the overall participants (*p* < 0.0001) as well as the group of full-participants (*p* < 0.0001), were significantly different from the Luxembourgish seniors (Chi-squared test).

The living situation (“single,” “in couple,” or “in cohabitation”) of the overall participants as well as the full-participants were very close to that of the Luxembourgish seniors (Fig 4A). The lifestyle distribution showed however a slight over- and under-representation of “life in couple” and “life in cohabitation” respectively, in the full and partial participants compared to Luxembourgish seniors (Fig 4B).

**Fig 5:**
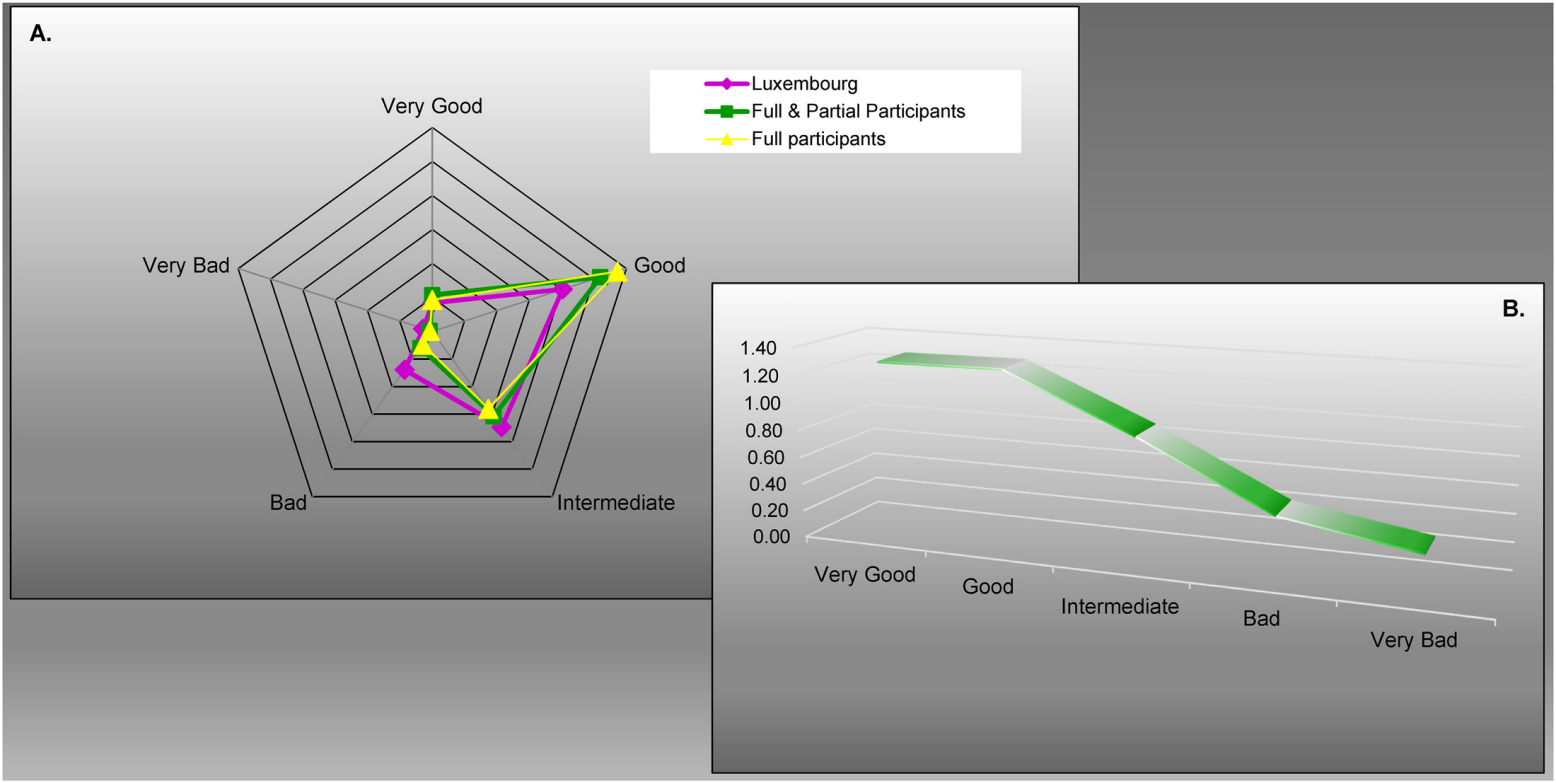
Answers supplied for “self-evaluation of health compared.” (A) Self-evaluation of health compared to people of similar age. (B) Representativeness depending on self-evaluation of health: the overall participants compared to the Luxembourgish seniors. From the full participants (*n* = 257), the overall participants (*n* = 391) and from the general population ≥ 65 years of Luxembourg (source: Eurostat, 2010, *n* = 69,976). The full participants as well as the overall participants were significantly different from the general population (*p* < 0.0001, Chi-squared test)

When asked “How do you perceive your health?”, the frequency of the response “intermediate” converged between populations. Positive answers (“good” and “very good”) were over-represented among the overall and full participants, and the negative responses (“bad” and “very bad”) were under-estimated, compared to Luxembourgish seniors (Fig 5A and 5B). We can speculate that participation in research studies is typically governed by either a concern about a specific disease, or conversely, the willingness for healthy individuals with few health concerns to be helpful. Here, we can assume the latter to be true, since we observed an over-representation of optimistic responses and an under-representation of pessimistic responses on the self-evaluation of health. Despite this, the distributions of responses were close between populations (Fig 5A).

### Determination of the prevalence of dementia and of cognitive complaints

The above-mentioned information collected through the short interviews from subjects who provided partial participation went some way to compensate for the low participation rate. Thus, the MemoVie study allowed us to estimate the prevalence estimates of dementia and of cognitive complaints for people aged over 64 in Luxembourg (Table 3).

**Table 3:** Prevalence estimates of dementia and cognitive complaints.

	Prevalence estimates	95% Confidence limits (CL)^#^
**Dementia**	3.8%	95%CL = [2.8–4.8]
**Cognitive complaints**	26.1%	95%CL = [17.8–34.3]

^#^: calculation obtained using sampling scheme and target population size

An estimates of 3.8% (95% Confidence limits (CL) = [2.8, 4.8], Table 3; Sample size: 1377, precision: 4%, Alpha risk: 5%) for the prevalence of dementia may seem relatively low in comparison to the data reported in other developed countries within a similar population (i.e., ≥ 65 years): 6.4% in Europe [21]; 7.1% in Latin America [28]; 8% in Canada [29]; 16.4% in the rural island town of Ama-Cho in Japan [30]. More generally, Sosa-Ortiz et al. [31] as well as Prince et al. [32] have reported a worldwide prevalence of 4.7%, a European one of 6.2%, and a 6.5% prevalence of dementia in the Americas (> 60 years). China [33], Africa, and Asia [31], have exhibited dementia rates of 3%, 2.6%, and 3.9% respectively (> 60 years). In their comparative approach of appraisal of dementia prevalence after 60 years, Prince et al. [32] mentioned a fourfold variation between world regions, in the global rates from 2.07% to 8.50% (even if most of estimates were concentrated between 5% and 7%). Altogether, these considerations allow us to assume that the dementia prevalence rate in Luxembourg we have reported lies within the band of rates of dementia prevalence estimates all over the world, even if low.

The possibility of underestimating prevalence estimates in this work has been explored and should now be discussed. First of all, the low participation may have impacted on the appraisal of prevalence, since the lowest dementia prevalence estimates come from surveys that report participation rates below 85% [34]. Secondly, we gathered dementia prevalence data using two different methods: 1) neuropsychological and medical examinations of the full-participants, and 2) information collected from families when a diagnosis of dementia had previously been made with certainty (for non- and partial participants). We could consequently assume that a formal diagnosis of dementia was not necessarily made for everyone who may have needed it. Indeed, it is well established that people with dementia (as well as with cognitive impairment, in general) are still under-diagnosed [35,36]. Wilkins and collaborators have correlated the lack of detection of dementia with, among other reasons, living alone [36]. The authors associated the presence of a spouse caregiver to the detection of the disease by primary care physicians. However, as previously mentioned, the living situation of the MemoVie full and partial participants was most commonly life as a couple, which may have minimized the under-detection of dementia.

To further assume that the non-participation group (constituting over two-thirds of the population), could drive downwards the obtained prevalence estimate, we evaluated the weighted prevalence for full participants only, with the same methodology (i.e. accounting for the sampling plan) as 4.66%, 95%CL = [2.10–7.22]. This prevalence is still lower than data reported in other developed countries. But here, the corresponding 95% confidence interval is much larger than the initial one [2.8–4.8] (describing an expected loss of power). In addition to the overlap in confidence intervals, it is worth noting that the prevalence estimate on full participants only, belongs to the confidence interval obtained on total participants.

**Fig 6:**
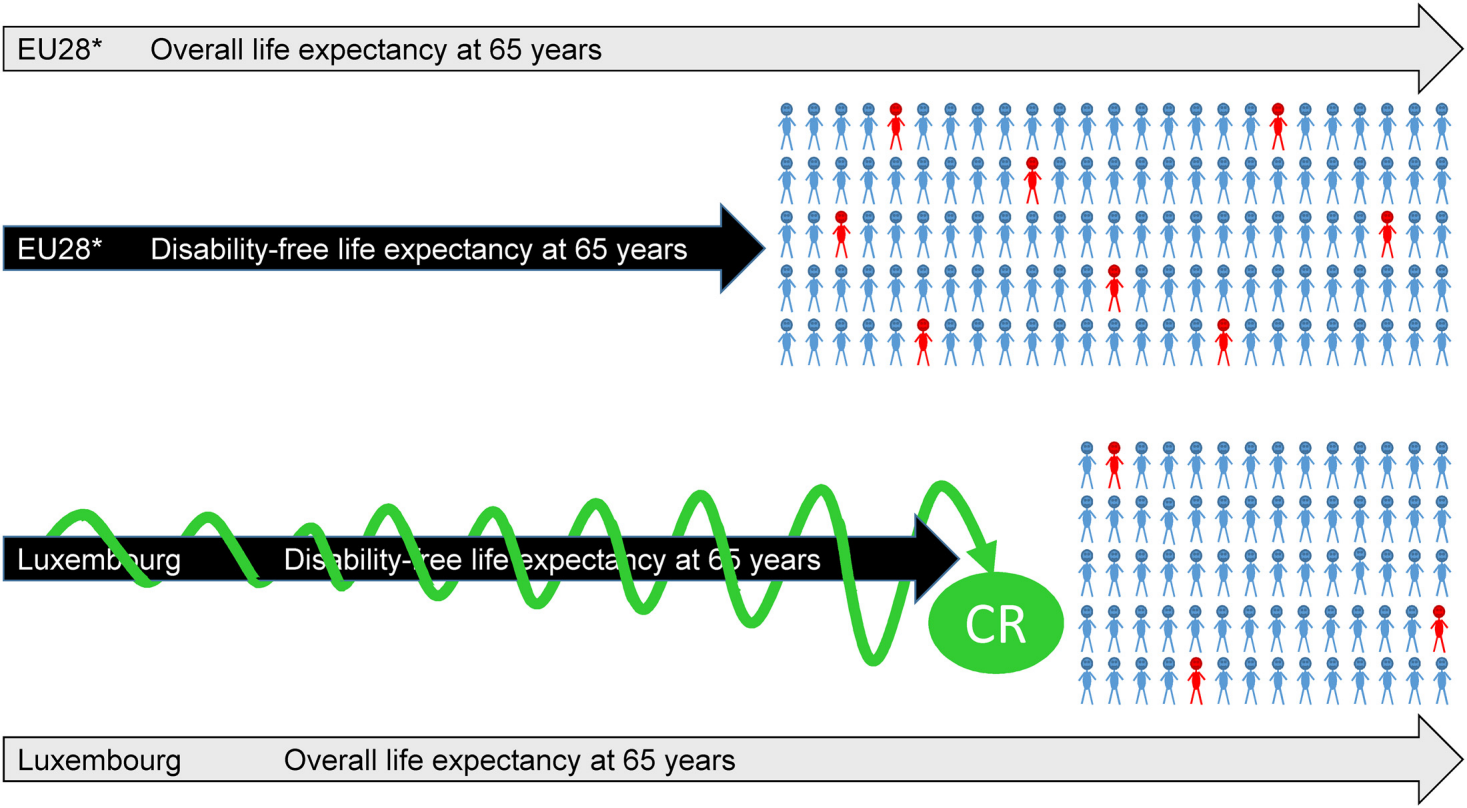
Luxembourg: The conjunction of a longer disability-free life expectancy at 65 years, a high cognitive reserve, and a low estimates of dementia?

However, beyond these considerations, the low prevalence estimates of dementia in Luxembourg inhabitants over the age of 64 years may also be explained by the considerable multilingualism, which is permanent and lifelong for residents. Consequently, the native population might acquire a higher level of cognitive reserve. In previous work on the MemoVie (full) participants, we showed that multilingualism is strongly associated with protection against cognitive impairment [8]. Other findings have shown that bilingualism also delays dementia [37,38]. The subsequent impact could thus be a reduction in the prevalence of this pathology, since mortality would occur before dementia onset for other reasons. Moreover, the low prevalence of dementia in Luxembourg could corroborate a longer disability-free life expectancy, since the indicator of "healthy life years at age 65" is 2.5 years greater, on average, for Luxembourg compared to 28 other European countries (2.2 years for men and 2.8 years for women, calculated for the total population over 5 consecutive years, 2008 to 2012 [39]). This favorable situation is illustrated in fig 6.

The other novel result of this work concerns the prevalence estimates of cognitive complaints, at 26.1% (CL = [17.8–34.3], Table 3; Sample size: 438, Precision: 4%, Alpha risk: 5%). As described earlier [40,41], cognitive complaints have been strongly correlated to forecasting of cognitive decline. Despite this assumption, the prevalence of cognitive complaints has rarely been the subject of field studies. Mewton et al. [42] registered a 33.5% prevalence of memory complaints in a community-dwelling Australian study of participants aged 65–85 years. Westoby and collaborators [41] reported a prevalence of cognitive complaints at ≥ 50 years of 46.5%, a rate that was 1.8-fold higher than the one we observed. Other studies have dealt with specific and small-sized groups [43]. Exploring and approaching memory complaints is faced with constraints that make reproducibility difficult [44,45].

**Fig 7:**
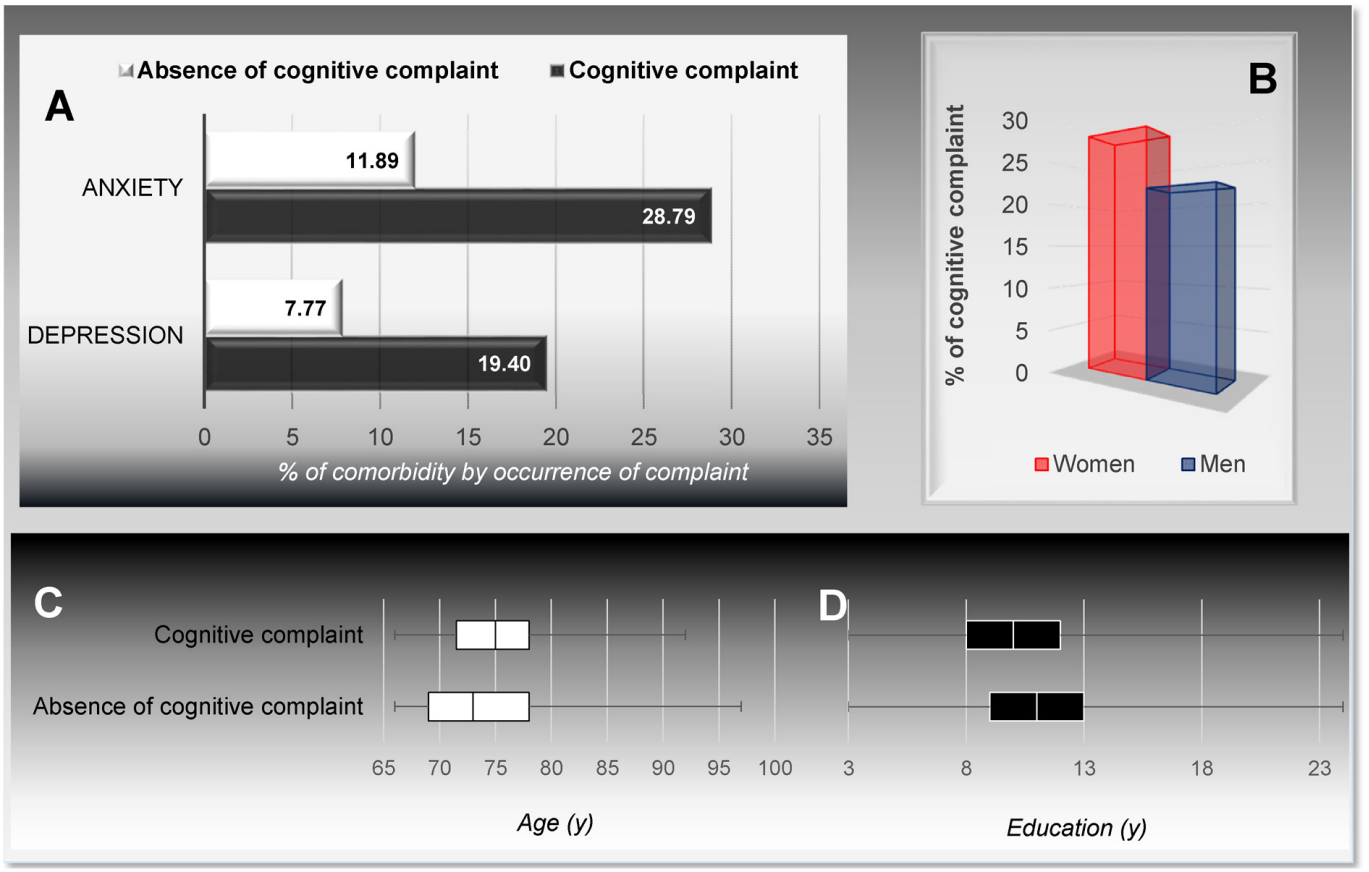
Association between cognitive complaints and anxiety or depression (A), gender (B), age (C), education (D). From the full participants: (A) Links between cognitive complaints and anxiety, as well as cognitive complaints and depression, respectively: *n* = 251, *p* = 0.001 and *n* = 260, *p* = 0.008, Chi-squared test. (B) Links between cognitive complaints and gender: *n* = 411, *p* = 0.11, Chi-squared test. (C) Links between cognitive complaints and age: *n* = 410, *p* = 0.01, Mann-Whitney test. (D) Links between cognitive complaints and education: *n* = 275, *p* = 0.02, Mann-Whitney test.

Objective cognitive complaints are known to be correlated with anxiety and depression [14,46,47], as are age and formal education [14,41,48]. Confirmatory analyses were performed to check this correlation in our population sample. As expected, depression and anxiety were significantly higher in people with cognitive complaints (almost 2.5-fold the rate observed in absence of cognitive complaints, Fig 7A). These complaints increased with age (significant mean age difference between those “with” and “without” complaints = 1.3 years; median difference = 2 years) and with shorter duration of education (significant mean duration difference between those “with” and “without” complaints = 1 year; median difference = 1 year) (Fig 7C and 7D). Finally, there was a trend for there to be a higher prevalence of cognitive complaints among women (Fig 7B), as has already been described by previous studies [14,41]. These associations with cognitive complaints are commonly accepted, and have been reproduced here, which make our data consistent and trustful.

Again, a low prevalence of cognitive complaints could be the consequence of a longer disability-free life expectancy of the Luxembourg population [39], insofar as memory cannot be disconnected from other health problems. Indeed, Montejo et al. [45] claim that an objective perception of memory is fostered by good health, quality of life, independence, and satisfying social relationships, and that illness, difficulties in daily living, dependency, and a bad quality of life are more likely to lead to health and memory complaints. In addition to the association with quality of life and activities of daily living, the same group of researchers showed that subjective memory complaints are also correlated with temporal orientation (93% of complaints among people displaying orientation problems, versus 22.2% among others) [14]. Moreover, a low prevalence of cognitive complaints may be the consequence of a higher cognitive reserve. High cognitive reserve through education and multilingualism was already observed in the Luxembourg population [8]. Consequently, we assessed this hypothesis among the subgroup of full-participants by considering education (as the number of years spent studying) and multilingualism (as the number of practiced languages). Only the first allowed us to corroborate the hypothesis of a significant association. Participants without cognitive complaints had a mean education duration of 1 year longer (11.52 ± 3.59) than did complaining participants (10.48 ± 3.64, *p* = 0.02, Fig 7D). We additionally found a trend concerning multilingualism, whereby we found 25.9% of complaints among multilingual subjects (who knew more than two languages) and 35.3% among non-multilingual subjects (*p* = 0.40, Fisher’s exact test).

## Conclusion

We set up a cohort study (called MemoVie) to be representative of seniors from Luxembourg. We analyzed groups according to their willingness to cooperate (full participation, partial participation, and non-participation).

Since the recruitment of the population was done from the IGSS register, based on age and gender, both of these variables are known for the expected MemoVie population i.e. for full-participants, partial participants, and non-participants. Individuals from these three levels of participation were not different in terms of gender. However, as often described, non-participants were older than full participants, and data from partial participants led us to suspect that they were also probably less educated. This study could not, however, provide information on the education of non-participants. Individuals who participate in research studies are usually significantly younger, with a better premorbid function and higher education than non-participants [49–52]. We also compared other parameters among these groups, such as lifestyle, dependency and disability, self-evaluated health, and cognitive complaints. Data obtained for full and partial participants were mostly similar (5 out of 8 of the considered parameters). In particular, the distribution of cognitive complaints was not statistically different between these two groups. “Cognitive complaints” was indeed the easiest parameter related to cognition that we could reliably measure in people reticent to provide full participation to a study on cognitive impairment. The parameters that differentiated both populations of participants (Lifestyle and Self-evaluation of health) were additionally explored by comparing their distribution in the MemoVie cohort and in the general Luxembourgish population (where national statistics were available), which showed a rather high level of similarity. Together, these investigations suggest that results from the MemoVie cohort are reliable and robust, and are close to the national prevalence estimates that could be obtained from the general population of seniors.

To our knowledge, our study is the first to assess the association between the prevalence of cognitive complaints and dementia in a general population exhibiting high cognitive reserve. Moreover, there are no existing data on national prevalence of dementia or on cognitive complaints in Luxembourg, which makes this work, an interesting contribution at both the national and international level. The relatively low prevalence of dementia and of cognitive complaints can be hypothesized to reflect a population with high cognitive reserve. Indeed, cognitive reserve, which is said to delay the onset of dementia, may also minimize cognitive complaints. This requires further research. If this is indeed the case, then our results highlight the necessity of an appropriate approach during ageing for individuals with high cognitive reserve.

## Supporting Information

S1 Dataset (XLSX)
